# Immunotherapy Plus Surgery Improves Survival in Microsatellite Instability-High Colon Cancer with Isolated Peritoneal Metastases

**DOI:** 10.3390/cancers17213496

**Published:** 2025-10-30

**Authors:** Daniel Aryeh Metzger, Yasmeen Chahal, Olivia Watman, Ying Li, Alessio Pigazzi, Despina Siolas, Mehraneh D. Jafari

**Affiliations:** 1 New York-Presbyterian—Weill Cornell, New York, NY 10065, USAdes2025@med.cornell.edu (D.S.); mdj9003@med.cornell.edu (M.D.J.); 2 New York-Presbyterian—Brooklyn Methodist Hospital, Brooklyn, NY 11215, USA; 3 City of Hope Orange County Lennar Foundation Cancer Center, Irvine, CA 92618, USA

**Keywords:** colon cancer, immunotherapy, metastases, surgery

## Abstract

**Simple Summary:**

Microsatellite instability-high (MSI-H) colon cancer is characterized by impaired DNA mismatch repair and robust responsiveness to immune checkpoint inhibition. However, the optimal management of patients with isolated peritoneal metastases (iPM), a site of spread associated with historically poor prognosis, remains poorly defined. Leveraging national real-world data, this study demonstrates that immunotherapy is associated with significantly improved survival compared to chemotherapy in patients with MSI-H colon cancer and iPM. Importantly, the combination of immunotherapy and surgical resection resulted in the greatest survival benefit. These findings challenge conventional paradigms that discourage surgery in metastatic disease and support a multimodal treatment strategy for this biologically distinct and clinically challenging patient population. The results highlight the need for prospective trials to define optimal sequencing and integration of surgery and immunotherapy in MSI-H colorectal cancer with peritoneal involvement.

**Abstract:**

Background: Microsatellite instability-high (MSI-H) colon cancer with isolated peritoneal metastases (iPM) represents a molecularly and anatomically distinct clinical subset with limited evidence to guide treatment. Given the unique immunogenic profile of MSI-H tumors and the historically poor prognosis of peritoneal dissemination, we evaluated the association of immunotherapy, chemotherapy, and surgery with survival outcomes in this population. Methods: Using the National Cancer Database, we identified patients with MSI-H colon cancer and iPM diagnosed between 2016–2021. Patients were stratified by systemic therapy type (immunotherapy, chemotherapy, combination) and surgical resection status. Kaplan–Meier and multivariable Cox regression analyses were used to assess overall survival (OS). Results: Among 598 patients, 22% received systemic treatment with immunotherapy and 76% underwent surgical resection. Immunotherapy was associated with significantly longer median OS compared to chemotherapy (33 vs. 18 months, *p* < 0.001). On multivariable analysis, immunotherapy remained independently associated with improved survival (HR: 0.46; *p* < 0.001). Surgical resection of the primary tumor with (HR: 0.40; *p* < 0.001) or without metastatectomy (HR: 0.41; *p* < 0.001) was associated with longer survival, and the combination of surgery and immunotherapy yielded the greatest survival benefit. Conclusions: Patients with MSI-H colon cancer and iPM treated with immunotherapy had significantly improved survival, compared to chemotherapy. Surgical resection combined with immunotherapy is associated with the greatest survival benefit, supporting a multimodal approach. These findings provide real-world evidence supporting integration of immunotherapy and surgery in this molecularly and anatomically distinct population.

## 1. Introduction

Colon cancer is among the most commonly diagnosed malignancies worldwide and remains a leading cause of cancer-related mortality. Ten percent of colon cancer patients present with peritoneal metastases, with nearly half of these cases involving isolated peritoneal metastases (iPM) [1]. Historically, systemic chemotherapy has served as the primary treatment modality, with cytoreductive surgery and heated intraperitoneal chemotherapy (HIPEC) reserved for select patients [2]. Despite aggressive multimodality approaches, outcomes for patients with peritoneal metastases remain poor, highlighting the need for novel and effective treatment strategies.

Microsatellite instability-high (MSI-H) tumors, present in approximately 10–20% of colon cancers, define a molecularly distinct subset characterized by defective DNA mismatch repair, high tumor mutational burden, and increased immune infiltration [3,4]. These tumors are often resistant to conventional chemotherapy but respond favorably to immune checkpoint inhibitors targeting PD-1/PD-L1 and CTLA-4 [5,6]. Accordingly, the National Comprehensive Cancer Network (NCCN) recommends immunotherapy as first-line therapy for patients with metastatic MSI-H colorectal cancer, including those with peritoneal involvement [7]. However, data supporting the survival benefits of immunotherapy in patients with MSI-H colon cancer and iPM are limited.

Systemic chemotherapy has historically shown limited efficacy in colorectal cancer patients with peritoneal metastases, and patients have worse survival when compared to patients with liver or lung metastases [8]. This may be due, in part, to the peritoneal–plasma barrier, which impedes the effective delivery of chemotherapeutic agents to peritoneal lesions [9,10]. In contrast, immune checkpoint blockade exerts systemic effects, activating cytotoxic T lymphocytes throughout the body, a mechanism that may remain effective even in poorly vascularized or compartmentalized sites like the peritoneum [11]. While the landmark KEYNOTE-177 trial demonstrated superior progression-free survival with pembrolizumab compared to chemotherapy in patients with MSI-H metastatic colorectal cancer, it did not stratify outcomes based on the site of metastasis [12]. Consequently, it remains unclear whether patients with isolated peritoneal involvement benefit from immune checkpoint blockade, particularly given the unique challenges of drug delivery to the peritoneal cavity.

Moreover, the role of surgery, especially primary tumor resection and metastasectomy, in the treatment of MSI-H patients with iPM is not well established. Existing literature suggests that surgical cytoreduction can improve survival in selected patients with peritoneal metastases, but the integration of surgery with modern immunotherapy in this biologically distinct subset has not been systematically studied [2]. Given these considerations, we conducted a population-based study to evaluate both systemic and surgical treatment strategies in patients with MSI-H colon cancer and iPM. We aimed to assess whether immunotherapy and/or surgery are associated with improved overall survival in this understudied subgroup. We aimed to evaluate ifimmunotherapy would confer a survival advantage compared to chemotherapy and that surgical intervention would further enhance outcomes, especially when integrated with immunotherapy in this molecularly defined and anatomically distinct subgroup.

## 2. Materials and Methods

We conducted a retrospective cohort study using data from the National Cancer Database (NCDB) to evaluate patients diagnosed with MSI-H colon cancer and iPM diagnosed between 2016 and 2021. Patients diagnosed after 2021 were not included in the main analysis since survival data is not yet available. Patients who did not receive systemic therapy were excluded. Variables collected included patient demographics (age, sex, race), comorbidity burden (Charlson–Deyo score), and treatment characteristics (facility type, primary tumor resection, metastatectomy, chemotherapy, and immunotherapy). Given the retrospective nature of the study, there was no randomization or blinding. Because the NCDB does not specifically code for iPM, we defined this population as patients with documented metastases at diagnosis but no recorded lung, liver, brain, or distant lymph node metastases, as previously published [13]. The NCDB *Immunotherapy* variable encompasses both immune checkpoint inhibitors and biologic agents such as anti-EGFR and anti-VEGF therapies. Since biologics are commonly administered with chemotherapy, while checkpoint inhibitors are commonly used as monotherapy, we treated patients coded as receiving both chemotherapy and immunotherapy as a separate “Chemotherapy + Immunotherapy” group to minimize misclassification.

We stratified patients into three groups for primary: Immunotherapy Alone (IO), Chemotherapy Alone (CT) and Chemotherapy + Immunotherapy (CT + IO). Group comparisons were made using the Wilcoxon rank-sum test for nonparametric continuous variables, Fisher’s exact test for categorical variables with small sample sizes or sparse data, and Pearson’s Chi-squared test for categorical variables with sufficient expected cell counts. All statistical tests were two-sided, and assumptions for each test were assessed where applicable.

Overall survival (OS) was the primary outcome. Kaplan–Meier analysis with log-rank testing was used to assess survival differences across treatment groups, and pairwise comparisons were conducted. An additional Kaplan–Meier analysis compared the Chemotherapy + Immunotherapy group to Immunotherapy Alone and Chemotherapy Alone.

Cox proportional hazards models were built using the entire cohort for time-to-event analysis after confirming proportionality assumptions. Univariate Cox regression identified clinicopathologic variables associated with OS. Multivariable Cox regression evaluated the association between treatment strategy and OS, adjusting for age, sex, Charlson–Deyo score, and histology. Hazard ratios (HRs) and 95% confidence intervals (CIs) were calculated. Lastly, using treatment data from 2016–2022, we evaluated trends in immunotherapy utilization by calculating the annual proportion of patients receiving immunotherapy among those diagnosed with MSI-H colon cancer and iPM. Patients with no available survival data were included in the utilization trend analysis.

A power calculation was not performed as all eligible patients in the database were included. All analyses were conducted using R version 4.2.0 (R Foundation for Statistical Computing, Vienna, Austria). The ‘gtsummary’ package was used for descriptive and regression tables, and survival analyses were performed using the ‘survival’ and ‘survminer’ packages.

## 3. Results

**Figure 1:**
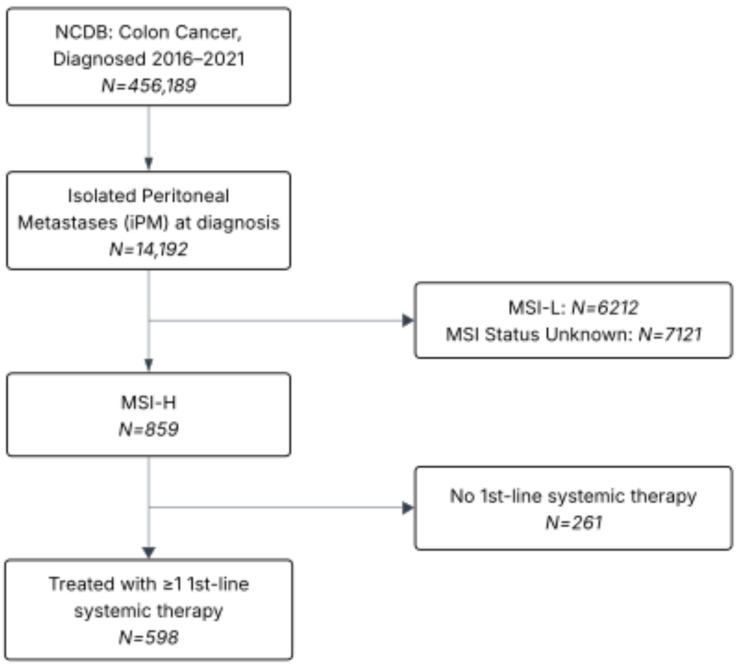
Inclusion/Exclusion Flow Diagram.

Of 456,189 patients in the NCDB diagnosed with colon cancer 2016–2021, 14,192 (3.1%) had isolated peritoneal metastases (iPM) at diagnosis. Among these, 859 (6.1%) had microsatellite instability–high (MSI-H) tumors. Of the MSI-H cohort, 598 (70%) received at least one line of systemic therapy (Figure 1).

A total of 598 patients with MSI-H colon cancer with iPM were identified in the NCDB. Of these, 132 (22%) received immunotherapy (IO) alone, 258 (43%) received chemotherapy (CT) alone and 208 (35%) a combination of chemotherapy and immunotherapy (CT + IO). Patients treated with IO were significantly older (75 vs. 67 years; *p* < 0.001) and had higher Charlson–Deyo comorbidity scores (1.8 vs. 1.4; *p* = 0.003) than those receiving chemotherapy alone. The IO cohort included a lower proportion of males compared to those treated with CT (36% vs. 40%; *p* = 0.006). No significant differences were observed in tumor grade or histology subtype. Patients treated with IO were less likely to undergo primary tumor resection compared to those receiving chemotherapy alone (42% vs. 51%; *p* = 0.002) (Table 1). Among patients who underwent surgery, systemic therapy was predominantly administered in the adjuvant setting (93%) with no significant difference across regimens (*p* = 0.2). Fewer than 10 patients in the cohort were treated with HIPEC.

Among patients treated with immunotherapy (Supplemental Table S1), there were no significant differences between those who underwent surgery and those who did not with respect to median age (75 vs. 77 years; *p* = 0.3), Charlson–Deyo score (*p* > 0.9), or treatment at an academic facility (63% vs. 64%; *p* = 0.9). Histologic subtype differed between groups, with mucinous adenocarcinoma more frequently observed among patients undergoing immunotherapy plus surgery compared to immunotherapy alone (27% vs. 7.9%; *p* = 0.03).

### 3.1. Survival Analysis

**Figure 2:**
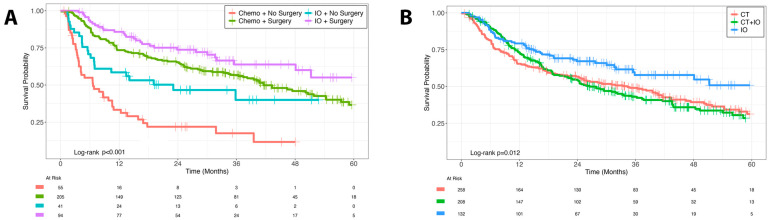
Kaplan–Meier Survival Analysis. (**A**). Overall Survival by Systemic and Surgical Treatment. (**B**). All Systemic Treatment Regimens.

Kaplan–Meier analysis (Figure 2A) demonstrated significantly improved survival among patients treated with immunotherapy and surgery (log-rank *p* < 0.001). The IO with surgery group had the highest 5-year OS (55.1%), with pairwise comparison demonstrating significantly improved survival compared to both IO without surgery (*p* = 0.001) and CT with Surgery (*p* = 0.035). IO without surgery also conferred superior survival compared to CT without surgery (*p* = 0.006), which had the poorest survival outcomes.

**Figure 3:**
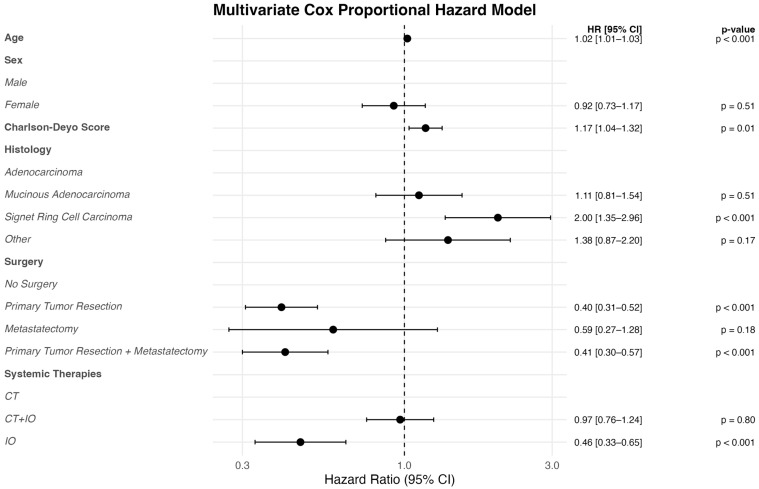
Multivariate Cox Proportional Hazard Model; Forest Plot.

Univariate analysis identified older age (HR: 1.02; 95% CI: 1.01–1.03; *p* < 0.001), higher Charlson–Deyo scores (HR: 1.23; 95% CI: 1.10–1.38; *p* < 0.001), and signet ring cell histology (HR: 1.99; 95% CI: 1.31–2.94; *p* < 0.001) were associated with worse OS. Components of treatment strategies were then evaluated in a multivariable model, adjusting for age, sex, Charlson–Deyo comorbidity index, and histology (Table 2, Figure 3). Systemic therapy with immunotherapy alone was associated with a 54% reduction in the risk of death compared to chemotherapy alone (HR: 0.46; 95% CI: 0.33–0.65; *p* < 0.001). Surgical resection of the primary tumor with metastatectomy (HR: 0.40; 95% CI: 0.31–0.52; *p* < 0.001) and without metastatectomy (HR: 0.41; 95% CI: 0.30–0.57; *p* < 0.001) were also associated with significantly reduced mortality. Metastatectomy alone (N = 16) showed a nonsignificant trend toward improved survival (HR: 0.59; 95% CI: 0.27–1.28; *p* = 0.20). Importantly, combination CT + IO therapy did not confer a survival advantage over CT alone, either by Kaplan–Meier analysis (5-year OS: 31.3% vs. 28.5%; *p* > 0.90; Figure 2B) or multivariable regression (HR: 0.97; 95% CI: 0.76–1.24; *p* = 0.80); Table 2).

### 3.2. Utilization Trends

**Figure 4:**
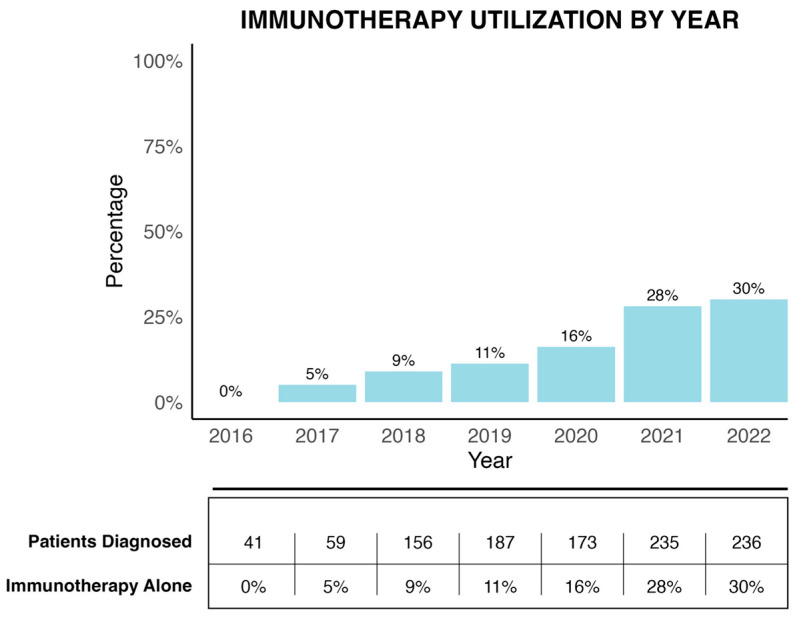
Immunotherapy Utilization by Year.

Annual case volume and immunotherapy utilization increased over the study period. The number of patients diagnosed with MSI-H colon cancer and iPM rose from 41 in 2016 to 236 in 2022. Concurrently, use of IO monotherapy rose from 0% to 30% (Figure 4), reflecting a broader shift in treatment paradigms following the adoption of immunotherapy in clinical guidelines.

## 4. Discussion

In this national cohort study of 598 patients with MSI-H colon cancer and iPM, we found that immunotherapy was associated with superior survival compared to chemotherapy, and that the combination of immunotherapy and surgery achieved the most favorable outcomes with a 5-year overall survival (OS) of 55.1%, suggesting a synergistic benefit from integrating surgical resection with immune checkpoint blockade in this unique clinical subset. On multivariable analysis, immunotherapy was associated with a 54% reduction in the hazard of death (HR 0.46), while primary tumor resection alone was associated with a 60% reduction (HR 0.40), and the combination of primary tumor resection and metastatectomy conferred a similar benefit (HR 0.41, 95% CI 0.30–0.57). These findings provide real world support for integrating surgical intervention into immunotherapy-based treatment strategies for patients with MSI-H colon cancer with iPM.

This is the first population-based study to directly compare immunotherapy to chemotherapy in this anatomically and molecularly distinct population. While the KEYNOTE-177 trial established checkpoint inhibition as a first-line option for patients with metastatic MSI-H colorectal cancer, it did not stratify outcomes by metastatic site. Smaller retrospective series, such as those by Saberzadeh-Ardestani et al. and Barraud et al., have reported responses to pembrolizumab immunotherapy in MSI-H patients with peritoneal involvement but were limited by sample size and lacked comparative treatment arms [14,15]. Our study addresses these limitations, offering population-level evidence for the effectiveness of immunotherapy specifically in patients with iPM.

The distinct biology of peritoneal metastases may account for the observed differential response to systemic therapies [16,17]. Conventional chemotherapy is considered less effective in peritoneal metastases compared to other metastatic sites, partly due to poor drug penetration across the peritoneal–plasma barrier [9,18]. Checkpoint inhibitors, by contrast, activate systemic immune responses, which may retain efficacy in poorly perfused or compartmentalized tumor sites like the peritoneum [11,19]. In addition, data from animal models suggest that immunotherapy induces a greater T cell response when administered prior to resection [20,21]. This immunologic mechanism may explain the significant survival benefit observed with immunotherapy in our cohort. Immunotherapy recipients in our study were older, had more comorbidities, and were less likely to undergo surgery, yet still had better survival outcomes, highlighting the superior efficacy of these agents even among higher-risk individuals. Immunotherapy utilization increased over the course of the study with a marked increase after the publication of the KEYNOTE-177 trial in 2020. However, immunotherapy remained underutilized at the end of the study period, with only 30% of eligible patients receiving it by 2022. Treatment at academic centers was associated with higher rates of immunotherapy use, suggesting disparities in access or variation in institutional expertise. This treatment gap likely reflects disparities in access, institutional expertise, and variability in the adoption of immunotherapy across practice settings.

The role of surgery in metastatic colon cancer, particularly with peritoneal involvement has long been debated. Randomized trials such as PRODIGE-7 demonstrated that complete cytoreductive surgery alone may yield durable survival in patients with limited peritoneal disease [2,22]. Likewise, the phase II CAIRO-6 trial established the safety and feasibility of integrating perioperative systemic therapy in patients undergoing CRS-HIPEC [23,24]. While neither study stratified by MSI status, retrospective data suggest that patients with MSI-H tumors may derive survival benefit from surgical approaches as their microsatellite stable counterparts [25,26]. Our study extends these findings, indicating that surgery retains clinical value, even in the era of immunotherapy and warrants further investigation as a potential standard approach for patients with MSI-H peritoneal metastases.

Nearly all patients in our cohort who underwent immunotherapy received it in the adjuvant setting or in the absence of surgery; fewer than ten patients were treated with neoadjuvant immunotherapy. While neoadjuvant checkpoint blockade is emerging as a promising strategy in early stage and locally advanced MSI-H tumors [27,28], the recent ATOMIC trial found improved disease-free survival with the addition of the checkpoint inhibitor atezolizumab to chemotherapy in the adjuvant setting for stage III MSI-H colon cancer [29,30]. Although that trial focused on non-metastatic disease, its results support a growing body of evidence that checkpoint blockade can meaningfully improve outcomes after curative-intent surgery. In our study, the subgroup of patients receiving both immunotherapy and surgery had the highest survival, suggesting that postoperative immunotherapy can extend benefit even in the metastatic setting. These data support a paradigm in which surgical cytoreduction with systemic immunotherapy may be a rational, multimodal approach for carefully selected patients with peritoneal-limited MSI-H disease. This study has limitations inherent to retrospective analyses including selection bias. Treatment selection was not randomized, and we lacked detailed data on specific agents, dosing, and duration. The NCDB does not capture progression-free survival, and disease-specific mortality. The coding of “immunotherapy” in NCDB includes biologic agents such as anti-EGFR or anti-VEGF antibodies, which are typically administered in combination with chemotherapy. In contrast, checkpoint inhibitors are frequently used as monotherapy. We addressed this by categorizing patients who received both chemotherapy and immunotherapy as a separate group. The absence of survival benefit in this subgroup compared to CT alone is difficult to interpret as mutational profiles that influence treatment response to these agents—such as RAS/NRAS, BRAF V600E, and HER2 status—are not captured in the NCDB and therefore could not be controlled for in our models.

Additionally, selection bias may have contributed to the observed survival benefit with surgery. While no significant differences were noted between immunotherapy + surgery and immunotherapy alone in terms of age or comorbidity scores, detailed measures of disease aggressiveness—such as Peritoneal Cancer Index (PCI)—were not available. Prospective studies with granular clinical and surgical data are needed to define the true benefit of surgery combined with immunotherapy in MSI-H peritoneal metastases.

Finally, while the ATOMIC trial supports immunotherapy after resection in stage III disease, the role of checkpoint inhibitors in the post-metastatectomy setting remains to be defined. Ongoing prospective studies should evaluate whether integrating immunotherapy with surgery can improve long-term outcomes in MSI-H iPM setting, as our findings suggest.

## 5. Conclusions

In summary, this study presents novel population-level evidence that combination immunotherapy and surgery are associated with improved survival in MSI-H colon cancer with isolated peritoneal metastases. These findings support an evolving treatment paradigm in which immunotherapy is not limited to unresectable cases but serves as a critical adjunct to surgery in patients with resectable iPM. Future trials should evaluate optimal sequencing, patient selection, and long-term outcomes of combined immunotherapy and surgical strategies in this molecularly defined population.

## Figures and Tables

**Table 1:** Cohort Characteristics.

Characteristic	Overall N = 598 ^1^	CT N = 258 ^1^	CT + IO N = 208 ^1^	IO N = 132 ^1^	*p*-Value ^2^
**Age**	67 (55, 76)	67 (56, 77)	61 (52, 71)	75 (63, 81)	<0.001
**Male**	259 (43%)	103 (40%)	108 (52%)	48 (36%)	0.006
**Race**					0.003
White	493 (84%)	218 (85%)	162 (80%)	113 (87%)	
Black	61 (10%)	28 (11%)	27 (13%)	6 (4.6%)	
Asian/Pacific Islander	26 (4.4%)	10 (3.9%)	7 (3.4%)	9 (6.9%)	
Other	9 (1.5%)	0 (0%)	7 (3.4%)	2 (1.5%)	
Unknown	9	2	5	2	
**Grade**					0.004
1	16 (4.0%)	11 (5.8%)	1 (0.8%)	4 (4.9%)	
2	159 (39%)	62 (33%)	59 (45%)	38 (46%)	
3	214 (53%)	105 (55%)	70 (53%)	39 (48%)	
4	14 (3.5%)	12 (6.3%)	1 (0.8%)	1 (1.2%)	
Unknown	195	68	77	50	
**Histology**					0.2
Adenocarcinoma	434 (73%)	183 (71%)	154 (74%)	97 (73%)	
Mucinous Adenocarcinoma	98 (16%)	40 (16%)	30 (14%)	28 (21%)	
Signet Ring Cell Carcinoma	36 (6.0%)	18 (7.0%)	15 (7.2%)	3 (2.3%)	
Other	30 (5.0%)	17 (6.6%)	9 (4.3%)	4 (3.0%)	
**Charlson–Deyo Score**					0.003
1	410 (69%)	196 (76%)	140 (67%)	74 (56%)	
2	111 (19%)	39 (15%)	42 (20%)	30 (23%)	
3	43 (7.2%)	11 (4.3%)	17 (8.2%)	15 (11%)	
4	34 (5.7%)	12 (4.7%)	9 (4.3%)	13 (9.8%)	
**Facility Type**					0.13
Community	35 (6.3%)	19 (7.9%)	10 (5.2%)	6 (4.9%)	
Comprehensive	207 (37%)	84 (35%)	84 (44%)	39 (32%)	
Academic	316 (57%)	139 (57%)	99 (51%)	78 (63%)	
Unknown	40	16	15	9	
**Surgery**					0.2
No Surgery	145 (24%)	54 (21%)	53 (25%)	38 (29%)	
Primary Tumor Resection	285 (48%)	131 (51%)	99 (48%)	55 (42%)	
Metastatectomy	16 (2.7%)	3 (1.2%)	7 (3.4%)	6 (4.5%)	
Primary Tumor Resection + Metastatectomy	152 (25%)	70 (27%)	49 (24%)	33 (25%)	
**Systemic/Surgery Timing**					0.2
Adjuvant	382 (91%)	178 (92%)	123 (87%)	>90% *	
Neoadjuvant	39 (9.3%)	15 (7.8%)	18 (13%)	<10% *	
Unknown	177	65	67	45	
**Margin Status**					**0.018**
No Surgery	145 (25%)	54 (21%)	53 (26%)	38 (30%)	
R+	104 (18%)	48 (19%)	45 (22%)	11 (8.7%)	
R0	324 (56%)	147 (58%)	101 (50%)	76 (60%)	
Surgery Performed, Margins Unknown	9 (1.5%)	6 (2.4%)	2 (1.0%)	1 (0.8%)	
**Surgical Approach**					0.10
No Surgery	142 (24%)	51 (20%)	53 (25%)	38 (29%)	
Minimally Invasive Approach	138 (23%)	54 (21%)	52 (25%)	32 (24%)	
Minimally Invasive Converted to Open	33 (5.5%)	21 (8.1%)	5 (2.4%)	7 (5.3%)	
Open	197 (33%)	93 (36%)	66 (32%)	38 (29%)	
Surgery Performed, Approach Unknown	88 (15%)	39 (15%)	32 (15%)	17 (13%)	
**Follow-Up (Months)**	24 (9, 40)	24 (7, 41)	23 (10, 42)	24 (13, 35)	0.8

^1^ Median (Q1, Q3); n (%); ^2^ Kruskal–Wallis rank sum test; Pearson’s Chi-squared test; Fisher’s exact test; * Counts less than 10 are not reported to protect patient privacy. Variables are bolded and variable levels are indented beneath when multiple levels are present.

**Table 2:** Cox Proportional Hazard Model: Overall Survival.

Variable	Univariable			Multivariable		
	HR	95% CI	*p*-Value	HR ^1^	95% CI	*p*-Value
**Age**	1.02	1.01, 1.03	**<0.001**	1.02	1.01, 1.03	**<0.001**
**Sex**						
Male	—	—		—	—	
Female	0.94	0.75, 1.17	0.6	0.92	0.73, 1.17	0.5
**Charlson–Deyo Score**	1.23	1.10, 1.39	**<0.001**	1.17	1.04, 1.32	**0.012**
**Facility Type**						
Community	—	—				
Comprehensive	0.88	0.56, 1.38	0.6			
Academic	0.73	0.46, 1.13	0.2			
**Histology**						
Adenocarcinoma	—	—		—	—	
Mucinous Adenocarcinoma	0.94	0.69, 1.29	0.7	1.11	0.81, 1.54	0.5
Signet Ring Cell Carcinoma	1.99	1.35, 2.94	**<0.001**	2.00	1.35, 2.96	**<0.001**
Other	1.52	0.96, 2.39	0.075	1.38	0.87, 2.20	0.2
**Grade**						
1	—	—				
2	1.11	0.54, 2.31	0.8			
3	1.33	0.65, 2.72	0.4			
4	1.03	0.39, 2.74	>0.9			
**Surgery**						
No Surgery	—	—		—	—	
Primary Tumor Resection	0.42	0.33, 0.55	**<0.001**	0.40	0.31, 0.52	**<0.001**
Metastatectomy	0.44	0.20, 0.94	**0.035**	0.59	0.27, 1.28	0.2
Primary Tumor Resection + Metastatectomy	0.41	0.30, 0.55	**<0.001**	0.41	0.30, 0.57	**<0.001**
**Systemic Therapies**						
CT	—	—		—	—	
CT + IO	1.01	0.80, 1.29	>0.9	0.97	0.76, 1.24	0.8
IO	0.64	0.46, 0.88	**0.006**	0.46	0.33, 0.65	**<0.001**
**Immunotherapy/Surgery Timing**						
Neo-Adjuvant	—	—				
Adjuvant	2.15	0.29, 15.8	0.5			

^1^ Adjusted for Age, Sex, Charlson–Deyo, Histology; Abbreviations: CI = Confidence Interval, HR = Hazard Ratio. Variables are bolded and variable levels are indented beneath when multiple levels are present.

## Data Availability

The data underlying this study were obtained from the National Cancer Database (NCDB). The NCDB is a joint project of the Commission on Cancer of the American College of Surgeons and the American Cancer Society. The data are not publicly available due to privacy and ethical restrictions but may be requested from the NCDB via their application process at https://www.facs.org/quality-programs/cancer/ncdb/ (accessed on 1 October 2024).

## Supplementary Materials

The following supporting information can be downloaded at: https://www.mdpi.com/article/10.3390/cancers17213496/s1, Supplemental Table S1. Cohort Characteristics: Immunotherapy With/Without Surgery.

## Abbreviations

The following abbreviations are used in this manuscript:

MSI-H	Microsatellite Instability-High
iPM	Isolated Peritoneal Metastases
IO	Immunotherapy Alone
CT	Chemotherapy Alone
CT + IO	Chemotherapy + Immunotherapy
HIPEC	Heated Intraperitoneal Chemotherapy
CRS-HIPEC	Cytoreductive Surgery with Heated Intraperitoneal Chemotherapy
OS	Overall Survival
HR	Hazard Ratio
CI	Confidence Interval
PD-1	Programmed Death-1
PD-L1	Programmed Death-Ligand 1
CTLA-4	Cytotoxic T-Lymphocyte Antigen 4
EGFR	Epidermal Growth Factor Receptor
VEGF	Vascular Endothelial Growth Factor
