# Supplementary material for: Immunotherapy Plus Surgery Improves Survival in Microsatellite Instability-High Colon Cancer with Isolated Peritoneal Metastases
Source: Cancers (Basel). 2025 Oct 30;17(21):3496. doi: 10.3390/cancers17213496 (PMC12609588; doi:10.3390/cancers17213496)
Supplement: Supplementary file 1 [file cancers-17-03496-s001.zip › cancers-3866086-supplementary.pdf]

Supplemental Table S1. Cohort Characteristics: Immunotherapy With/Without Surgery.

Characteristic	IO + No Surgery N = 38 [1]	IO + Surgery N = 94 [1]	*p*-Value [2]
**Age**	77 (65, 85)	75 (62, 81)	0.3
**Male**	14 (37%)	34 (36%)	>0.9
**Grade**			0.6
1	0 (0%)	4 (5.0%)	
2	0 (0%)	38 (48%)	
3	2 (100%)	37 (46%)	
4	0 (0%)	1 (1.3%)	
Unknown	36	14	
**Histology**			0.03
Adenocarcinoma	33 (87%)	64 (68%)	
Mucinous Adenocarcinoma	3 (7.9%)	25 (27%)	
Signet Ring Cell Carcinoma	0 (0%)	3 (3.2%)	
Other	2 (5.3%)	2 (2.1%)	
**Charlson–Deyo Score**			>0.9
1	23 (61%)	51 (54%)	
2	8 (21%)	22 (23%)	
3	4 (11%)	11 (12%)	
4	3 (7.9%)	10 (11%)	
**Facility Type**			0.9
Community	1 (2.8%)	5 (5.7%)	
Comprehensive	12 (33%)	27 (31%)	
Academic	23 (64%)	55 (63%)	

[1] Median (Q1, Q3); *n* (%); [2] Wilcoxon rank sum test; Pearson's Chi-squared test; Fisher's exact test. Variables are bolded and variable levels are indented beneath when multiple levels are present.
